# Nonbiological factors affecting outcomes in adolescents and young adults with lymphoma

**DOI:** 10.3389/fpubh.2023.1261066

**Published:** 2023-10-18

**Authors:** Aditi Dhir, Narendranath Epperla, Luciano J. Costa, Ana C. Xavier

**Affiliations:** ^1^Department of Pediatrics, Division of Hematology/Oncology, University of Miami Miller School of Medicine, Miami, FL, United States; ^2^Department of Medicine, Division of Hematology, The James Cancer Hospital and Solove Research Institute, The Ohio State University, Columbus, OH, United States; ^3^Department of Medicine, Division of Hematology and Medical Oncology, University of Alabama at Birmingham, Birmingham, AL, United States; ^4^Department of Pediatrics, Division of Hematology/Oncology, University of Alabama at Birmingham, Birmingham, AL, United States

**Keywords:** socioeconomic, health disparities, adolescent and young adult (AYA) cancer, lymphoma, non-Hodgkin’s lymphoma, Hodgkin (cHL)

## Abstract

The impact of nonbiological factors (NBF) on survival was investigated in a large cohort of adolescents and young adults (AYA) with lymphoma in the United States (US). We found that uninsured and Medicaid AYA beneficiaries with classical Hodgkin lymphoma (cHL) and non-Hodgkin lymphoma (NHL) are at significantly increased risk of death when compared with their insured counterpart even after adjustment for other factors affecting survival. Increased risk of death was also noted for Non-Hispanic Black (NHB) patients with cHL and NHL when compared to Non-Hispanic White (NHW) patients, however, only Hispanic patients with NHL were found to have a significantly increased mortality risk while those with cHL were not. NHL AYA patients residing in lower-income counties are at increased risk of death. The strong association of NBF with survival indicates opportunities to improve the survival of AYA lymphoma patients by improving access/quality of care in the US.

## Introduction

Lymphomas are common in adolescents and young adults (AYA; age 15–39 years). In the United States (US), classic Hodgkin lymphoma (cHL) and non-Hodgkin lymphoma (NHL) comprise up to 30% of all cancers among AYA (1). However, little change in survival of AYA lymphoma patients has been noted over the years as compared to their younger counterparts (2). Outcomes have also been improving among older adults with lymphomas at a faster rate than in AYA (2). There is notable variation in biological characteristics of lymphoma that account for the differences in outcomes across age groups. Clinical characteristics such as histology, stage at presentation, age, and sex, unique lymphoma gene expression profiles, and other molecular features have been shown to influence response to treatment and outcomes (3, 4).

However, other challenges faced by AYA lymphoma patients, including wide variability in access to medical care, varying therapeutic approaches based on age, location or provider, and/or access to therapeutic clinical trials may also be contributing to outcomes in this population (5, 6). Nonbiological factors (NBF) such as race/ethnicity, insurance status, education, and income have been shown to affect survival in cancer (7, 8). Similar studies for older patients with lymphoma have been undertaken revealing disparities in outcomes based on insurance, income and socioeconomic status (SES) (9, 10). In this study, we aimed at determining the impact of NBF on the survival outcomes in a large cohort of AYA patients with lymphoma in the US.

## Methods and results

### Data source

We utilized data from the National Cancer Institute (NCI) Surveillance, Epidemiology, and End Results (SEER-18) registry. The SEER-18 includes Atlanta, Connecticut, Detroit, Hawaii, Iowa, New Mexico, San Francisco-Oakland, Seattle-Puget Sound, Utah, Los Angeles, San Jose-Monterey, Rural Georgia, Alaska Native, Greater California, Kentucky, Louisiana, New Jersey, and Greater Georgia registries, corresponding to 27.8% of the US population. We extracted data from individuals 15–39 years of age, registered with a new diagnosis of lymphoma during the years of 2007 and 2014. Data collection was started in 2007 since that was when insurance information became available in the registry.

We included all cases of *de novo* cHL (International Classification of Diseases for Oncology, 3rd Edition [ICD-O-3] codes 9650/3, 9663/3, 9651/3, 9652/3, and 9653/3) and for NHL codes 9680/3 (diffuse large B-cell lymphoma), 9687/3 (Burkitt lymphoma), 9819/3, 9837/3 (lymphoblastic lymphoma), and 9714/3 (anaplastic large cell lymphoma). The ICD-O-3 reflects the cHL and NHL categories outlined in the 2008 revision of the World Health Organization (WHO) classification (11). Individual case information was retrieved using the case list function of the Surveillance Research Program, NCI SEER*Stat software^1^ version 8.1.5. Survival follow-up was available to the end of 2014. We did not include cases in which the only information source was the death certificate or autopsy report.

We extracted age, sex, stage (localized, advanced), race/ethnicity category [Hispanic, Non-Hispanic Black (NHB), Non-Hispanic White (NHW), other], year of diagnosis, ICD-O-3 code, county of residency, insurance status (insured, Medicaid, uninsured), median household income in the county of residency, educational achievement in the county of residency, duration of follow-up, and vital status for each individual.

### Statistical analysis

Survival analysis for each insurance category (insured, uninsured, Medicaid) was calculated using the Kaplan–Meier method. The impact of NBF (race/ethnicity, insurance status, median household income, and educational achievement in the county of residency) along with biological factors (histology, stage, sex, age,) on survival of AYA lymphoma patients was analyzed using Cox proportional hazards model. Variables were chosen using a stepwise backward process with a probability of entry of 0.05 and a probability of removal of 0.10. Continuous variables with no linear correlation with outcome (median household income in the count of residency, educational achievement in the county of residency) were classified in quintile or tiers. All statistical analysis was performed using SPSS statistical software (Version 22.0; IBM Corporation, Armonk, NY). In all inference analyses, two-sided *p* values <0.05 were considered to indicate statistical significance.

### Non-biological factors

**Figure 1:**
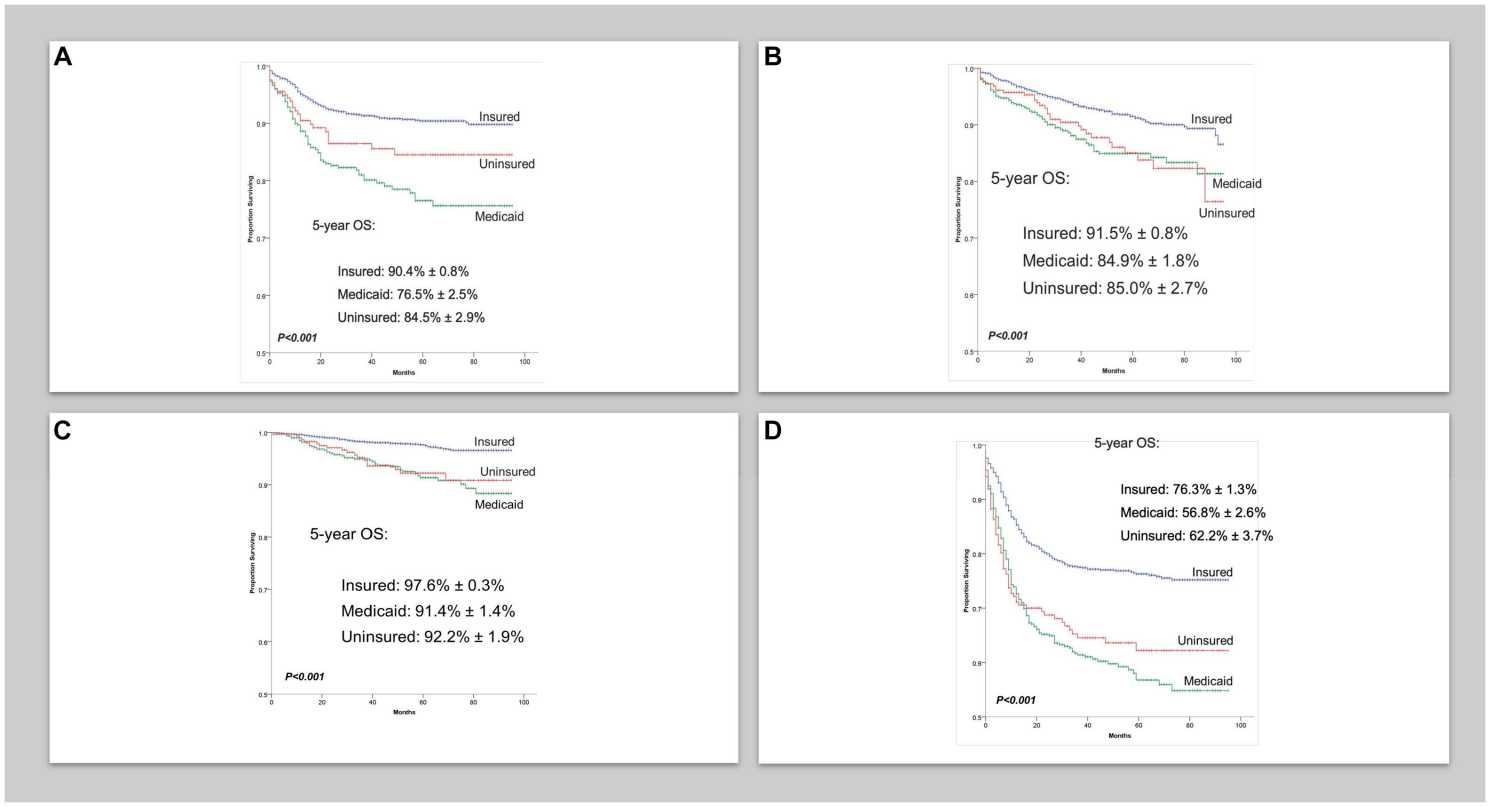
**(A)** Overall survival in localized classical Hodgkin lymphoma cases per insurance status. **(B)** Overall survival in advanced classical Hodgkin lymphoma cases per insurance. **(C)** Overall survival in localized non-Hodgkin lymphoma cases per insurance status. **(D)** Overall survival in advanced non-Hodgkin lymphoma cases per insurance status.

There were 8,173 AYA cases of cHL and 4,973 AYA cases of NHL with a median follow-up of 44 (0–95 months) and 32 months (0–95 months), respectively. The characteristics of patients are shown in Table 1. Five-year overall survival (OS) for AYA patients with localized cHL according to insurance status was 98% vs. 91% vs. 92% for insured, Medicaid, and uninsured patients, respectively (*p* < 0.001; Figure 1A). The 5-year OS for AYA patients with advanced stage cHL was 91.5% vs. 85% vs. 85% for insured, Medicaid, and uninsured patients, respectively (*p* < 0.001; Figure 1B). In multivariate analysis, the increased risk of death was associated with Medicaid [Hazard ratio (HR) 2.23. 95% confidence interval (CI) 1.76–2.81, *p* < 0.001], uninsured status (HR 1.88, 95% CI 1.38–2.55, *p* < 0.002), and NHBs (HR 2.03, 95% CI 1.59–2.60, *p* < 0.001) after adjustment for age, sex, histology, and stage (*p* < 0.001; Table 2).

**Table 1:** Classical Hodgkin and Non-Hodgkin Lymphoma cohorts characteristics.

Classical Hodgkin lymphoma cohort		Non-Hodgkin Lymphoma cohort	
	*N* = 8,173		*N* = 4,973
FU, months (range)	44 (0–95)	FU, months (range)	32 (0–95)
Age in years, median	28.5	Age in years, median	27
Male sex (%)	4,114 (50.4)	Male sex (%)	3,101 (62.4)
Race (%)		Race (%)	
Non-Hispanic Black	1,175 (14.4)	Non-Hispanic Black	731 (14.7)
Non-Hispanic White	5,062 (61.9)	Non-Hispanic White	2,619 (52.7)
Hispanic	1,489 (18.2)	Hispanic	1,126 (22.6)
Other	360 (4.4)	Other	456 (9.2)
Unknown	87 (1)	Unknown	41 (0.8)
Histology subtype (%)		Histology subtype (%)	
Classical NOS*	1,583 (19.4)	Diffuse Large B-cell	3,583 (72)
Nodular sclerosis	5,724 (70)	Burkitt	648 (13)
Mixed cellularity	660 (8)	Lymphoblastic	398 (8)
Lymphocyte-rich	156 (2)	Anaplastic large cell	344 (7)
Lymphocyte-depleted	51 (0.6)		
Stage, localized (%)	4,962 (60.7)	Stage, localized (%)	
Insurance status		Insurance status	
Insured	5,876 (71.9)	Insured	3,325 (66.8)
Medicaid	1,401 (17.2)	Medicaid	1,065 (21.5)
Uninsured	639 (7.9)	Uninsured	438 (8.8)
Unknown	257 (3.1)	Unknown	145 (2.9)

*NOS, not otherwise specified.

**Table 2:** Factors associated with increased risk of death in classical Hodgkin lymphoma.

	HR (95%)	*P*
Age at diagnosis	1.03 (1.01–1.04)	<0.001
Male sex	1.34 (1.09–1.64)	0.005
Race/ethnicity		
Non-Hispanic White	1 (reference)	
Non-Hispanic Black	2.03 (1.59–2.60)	<0.001
Non-Hispanic Other	1.11 (0.72–1.71)	0.65
Hispanic	1.13 (0.86–1.49)	0.38
Histology subtypes		
Nodular sclerosis	1 (reference)	
Classical NOS	1.34 (1.06–1.70)	0.016
Mixed cellularity	1.35 (0.98–1.87)	0.06
Lymphocyte-rich	0.53 (0.2–1.42)	0.21
Lymphocyte-depleted	2.09 (0.93–4.73)	0.08
Stage		
Localized	1 (reference)	
Advanced	2.51 (2.03–3.10)	<0.001
Insurance status		
Insured	1 (reference)	
Medicaid	2.23 (1.76–2.81)	<0.001
Uninsured	1.88 (1.38*2.55)	<0.002
Unknown	1.46 (0.82–2.58)	0.20

*NOS, not otherwise specified.

Five-year OS for AYA patients with localized NHL according to insurance status was 90% vs. 76.5% vs. 84.5% for insured, Medicaid, and uninsured patients, respectively (*p* < 0.001; Figure 1C). The 5-year OS for advanced stage NHL was 76% vs. 57% vs. 62% for insured, Medicaid, and uninsured patients, respectively (*p* < 0.001; Figure 1D). In multivariate analysis, the increased risk of death in AYA NHL was associated with Medicaid (HR 1.95, 95% CI 1.67–2.27, *p* < 0.001), uninsured status (HR 1.48, 95% CI 1.18–1.84, *p* = 0.001), NHB (HR 1.68, 95% CI 1.41–2.01, *p* < 0.001) and Hispanics (HR 1.29, 95% CI 1.08–1.54, *p* = 0.004) when compared to the NHW counterparts, and county level of income in the second (HR 1.33, 95% CI 1.07–2.64), third (HR 1.54, 95% CI 1.25–1.90), and fourth lower quartiles (HR 1.57, 95% CI 1.27–1.93) (*p* < 0.001), after adjustments for age, sex, histology, and stage (Table 3).

**Table 3:** Factors associated with increased risk of death in non-Hodgkin lymphoma.

	HR (95%)	*P*
Age at diagnosis	1.03 (1.02–1.04)	<0.001
Male sex	1.52 (1.30–1.77)	<0.001
Race/ethnicity		
Non-Hispanic White	1 (reference)	
Non-Hispanic Black	1.68 (1.41–2.01)	<0.001
Non-Hispanic Other	1.18 (0.90–1.53)	0.25
Hispanic	1.29 (1.08–1.54)	0.004
Year of diagnosis	0.93 (0.91–0.97)	<0.001
Histology subtypes		
Diffuse large B-cell lymphoma	1 (reference)	
Burkitt lymphoma	1.45 (1.21–1.74)	<0.001
Lymphoblastic lymphoma	1.68 (1.35–2.10)	<0.001
Anaplastic large cell lymphoma	1.45 (1.12–1.88)	0.006
Stage		
Localized	1 (reference)	
Advanced	2.34 (2.01–2.72)	<0.001
Income quartile		
First	1 (reference)	
Second	1.33 (1.07–1.64)	0.01
Third	1.54 (1.24–1.90)	<0.001
Fourth	1.57 (1.27–1.93)	<0.001
Insurance status		
Insured	1 (reference)	
Medicaid	1.95 (1.67–2.27)	<0.001
Uninsured	1.48 (1.18–1.84)	0.001
Unknown	1.07 (0.66–1.72)	0.79

## Discussion

Uninsured and Medicaid AYA beneficiaries with cHL and NHL are at significant increased risk of death when compared with their insured counterpart even after adjustment for other factors affecting survival. Parikh et al. utilized the National Cancer Database (NCDB) to conduct a retrospective study of HL patients (including older patients up to 90 years of age) diagnosed between 1998 and 2011 in the US. Similar to our results, they also reported an inferior OS for patients with unfavorable insurance (Medicaid/uninsured) as compared to the favorably insured (5-year OS 54% vs. 87%, respectively, *p* < 0.01) (9). In Ohio alone, US residents aged 15–54 years diagnosed between 1996 and 2002, survival was unfavorable for Medicaid vs. non-Medicaid patients with different cancer types (12). There was a greater proportion of patients with cHL or NHL with Medicaid than non-Medicaid. The authors further showed strikingly differences in survival in the Medicaid peri/post-diagnosis group (defined as patients enrolled in Medicaid in the 3-month window before, upon or after cancer diagnosis) in comparison to patients in the Medicaid pre-diagnosis (defined as patients enrolled in Medicaid at least 3 months before cancer diagnosis) for cHL patients, but not for NHL patients. Importantly, after adjusting for patients’ demographics, marital status, census tract income and educational attainment, county of residence, and cancer site, Medicaid patients were significantly more likely to experience an unfavorable survival outcome (13).

By potentially leading to delays in diagnosis and advanced stage at presentation, lack of access to appropriate treatment, inability to obtain supportive care, and inadequate health coverage are increasingly becoming a national concern as it directly effects the overall cost of care. In addition to lack of insurance, difficulties in transitioning from pediatric to adult care settings and lack of knowledge in preventable health care measure are some of the leading reasons why young adults have the lowest rates of health care utilization (6).

Per the most recent census data, it is estimated that 28 million Americans are uninsured, of which majority are 19- to 64 years old, have less than a high school education and/or lower incomes.^2^ Our study found that AYA NHL patients residing in lower income counties are also at increased risk of death. Likewise, a Danish NHL study showed lower survival rates for individuals with lower income and education levels (10). The impact of income is replicated even more drastically among high, middle, and low income nations with cure rates of mature B-cell lymphoma ranging from 20 to 70% in residents of low and middle income countries as compared to 90% for those residing in high-income countries (12).

As reported in the literature, the overwhelming majority of patients with cHL and NHL were NHWs and similar to prior studies, black race was a predictor of worse survival (14–17). On the contrary, a study investigating race/ethnic differences in patients with diffuse large B-cell lymphoma (DLBCL), a major NHL subtype, noted similar risk of mortality for NHBs and NHWs and a survival benefit for Hispanic patients as compared to NHWs (18). This study was however limited to a single institution with mostly older patients (mean age > 50 years), while Berkman et al. (17) reported disparities only for AYA patients with DLBCL and noted significantly worse survival for NHB patients (17).

Literature from other countries also emphasize the role of other NBF in outcomes for lymphoma. A population-based study in France showed that unmarried patients have lower survival as compared to married patients with DLBCL (19). After adjustments for potential confounders, lower SES remained independently associated with poorer survival in a Brazilian cHL cohort analysis (20). Data from the Swiss cancer registry reported a favorable association between high SES and parent education for children with lymphomas. Interestingly, they did not find the same association for pediatric leukemia patients, potentially highlighting the importance of standardized treatment approaches (7).

The data on treatment received including chemotherapy agents used, radiation therapy use, medication compliance, duration of treatment and could not be accessed given nature of the SEER database and represents a limitation of our analysis. Furthermore, the setting of treatment, academic medical center with clinical trial availability as compared to smaller community hospital, could not be analyzed. Misclassification of ethnicity and cancers in registry records is also possible. Variables such as Zip code were not analyzed which could provide insights into potential spatial dependencies or regional disparities in survival outcomes.

However, our data highlights the need to account for socioeconomic factors when studying outcomes in AYA patients with lymphoma. Understanding that lack of insurance or Medicaid insured status and lower income are strongly associated with increased mortality in AYA lymphoma patients, indicates opportunities to improve survival by improving access and quality of care in the US. In addition, recognizing the race/ethnic disparities that exist can help pave way for further studies which are necessary to understand the association of socioeconomic determinants of health and survival in lymphoma.

## Data availability statement

Publicly available datasets were analyzed in this study. This data can be found here: National Cancer Institute (NCI) Surveillance, Epidemiology, and End Results (SEER-18) registry.

## Author contributions

AD: Conceptualization, Formal analysis, Methodology, Writing – original draft, Writing – review & editing. NE: Conceptualization, Formal analysis, Methodology, Writing – original draft, Writing – review & editing. LC: Conceptualization, Formal analysis, Methodology, Writing – original draft, Writing – review & editing. AX: Conceptualization, Formal analysis, Methodology, Writing – original draft, Writing – review & editing.

## Figures and Tables

**Table:** 

AYA	Adolescent and Young Adult
cHL	Classic Hodgkin lymphoma
CI	Confidence Interval
DLBCL	Diffuse large B-cell lymphoma
HR	Hazard ratio
ICD-O	International Classification of Diseases for Oncology
NBF	Nonbiological Factors
NCDB	National Cancer Database
NCI	National Cancer Institute
NHL	Non-Hodgkin lymphoma
OS	Overall Survival
SES	Socioeconomic status
NHB	Non-Hispanic Black
NHW	Non-Hispanic White
SEER	Surveillance Epidemiology and End Results
US	United States

## Footnotes

^1^seer.cancer.gov/seerstat/

^2^ census.gov
